# Inhibitory control impairments underlie associative memory deficits in posttraumatic stress disorder

**DOI:** 10.1371/journal.pone.0329810

**Published:** 2025-08-14

**Authors:** Jonathan Guez, Rotem Saar-Ashkenazy, Eldad Keha, Hadar Shalev

**Affiliations:** 1 Department of Psychology, Achva Academic College, Beer-Tuvia Regional Council, Israel; 2 Beer-Sheva Mental Health Center, Beer-Sheva, Israel,; 3 Faculty of Social Work, Ashkelon Academic College, Ashkelon, Israel; 4 Department of Psychiatry, Soroka University Medical Center, Beer-Sheva, Israel; Taipei Veterans General Hospital, TAIWAN

## Abstract

**Objective:**

Posttraumatic-stress disorder (PTSD) patients suffer from cognitive dysfunction and show impairments even in non-trauma-related memory. Research has focused on the relationship between associative-memory and PTSD severity due to patients’ tendency to over-generalize from traumatic cues to neutral ones, leading to escalation of traumatic symptoms. In this study we aim to test to what extent inhibitory control impairments are correlated to associative-memory deficits in PTSD.

**Method:**

Twenty PTSD and 22 control participants were included. Posttraumatic symptoms were assessed via a board-qualified psychiatrist and the Post-Traumatic Diagnostic Scale. Inhibitory abilities were evaluated using the anti-saccade task and memory performance was probed using a words/pictures item-association paradigm.

**Results:**

Generally, PTSD patients performed lower than controls in both tasks. Lower associative-memory performance was observed in posttraumatic patients and was attributed to increased false-alarm rate in this group. In addition, we observed a strong significant positive correlation between associative pictorial memory performance and inhibitory performance, and in accordance, a significant negative correlation between the number of false-alarm responses in the associative pictorial test and inhibitory performance in the PTSD group.

**Conclusions:**

These results support the hypothesis that inhibitory control impairments are associated with (pictorial) associative-memory deficits in PTSD.

## 1. Introduction

Posttraumatic-stress disorder (PTSD) may develop in response to an acute traumatic event and is manifested in re-experiencing, avoidance, negative alterations in cognition and mood and hyper-arousal [1]. Individuals with PTSD present mental, physical, emotional and cognitive dysfunction [2–4]. Notwithstanding, PTSD patients present not only trauma-related bias/impairments [5]; For example, PTSD has been reported to be associated with cognitive abnormalities in processing both emotional information that is relevant to their traumatic source and emotionally neutral information which is irrelevant to their traumatic experience [6]. Notably, PTSD patients were reported to show robust memory impairments, regardless of emotional context [7]. Among the reported impairments are immediate and delayed recall/recognition of verbal and visual-spatial information, working memory and executive memory [8–18].

Of special interest is the relationship between traumatic symptoms and associative memory. Researchers have focused on this relationship due to two main tendencies of PTSD patients; The first, to over-generalize from traumatic cues to neutral ones and the second, to show elevated false-alarms (FA) responses that are correlative to their traumatic symptoms. Reviewing studies exploring memory in PTSD, reveals heterogeneous results. Some studies did not report significant differences in performance between healthy controls and traumatized participants [19] or those with PTSD [20], whereas others showed trends toward reduced associative performance [21]. A different study demonstrated impaired memory for neutral words [22]. Associative memory deficits for neutral stimuli were also reported in acute stress disorder (ASD; [23]) and chronic PTSD patients [24–26] and were attributed to an increase in wrong association of unrelated items (i.e., FA responses). The difference between item and associative recognition can be explained through the prism of the dual-process theory, asserting that single and associated stimuli are handled differently [27]. The dual-process theory highlights two processes that underlie memory: familiarity and recollection. Familiarity is a process that is considered automatic and thus involves the recognition of information in the absence of specific details. Recollection however is a process which involves executive functioning and requires recognition of specific situations/events that are accompanied by specific details. The difference between familiarity and recollection is often tested by using item-association memory paradigms. Results from these paradigms suggest that familiarity may be sufficient for correct recognition of single items; however correct recognition of associations is based on recollection processes [28]. In this manner, successful associative recognition cannot benefit from familiarity processes; rather it depends on recollection processes requiring that enough specific details be retained in order to decide whether the presented items were presented/not presented together during learning. This decision involves both high cognitive load and high executive control. Studies have reported impaired executive functioning/control in PTSD [29]; these include deficits in attention, voluntary allocation of attentional recourses as well as sustained attention, working memory, strategic planning behavior, strategic switching behavior and inhibitory functions [30,31].

Inhibitory control is considered a form of cognitive control that enables the suppression of irrelevant/inappropriate information or an automatic response in a situation that requires a novel response [32,33]. As in the case of associative memory, relatively few studies have examined responses to attentional tasks engaging cognitive and inhibitory control using both neutral and affective stimuli in PTSD; these studies also show heterogeneous results. For example, using neutral stimuli, it has been reported that PTSD patients showed significantly poorer results on both objective (Color-Word Interference Test, CWIT) and subjective (self-report questionnaire) measures of inhibitory control as compared to trauma-exposed and control participants [34]. Other studies employing an emotional Stroop task reported attentional bias in PTSD (mostly expressed in longer response time, RT) to threat/trauma-related stimuli, but less so for threat-free and neutral stimuli [35–38]. However, some researchers found brain differences in the anterior cingulate and visual cortex between PTSD participants and healthy controls, but did not find evidence for slower RTs in trauma-related versus neutral words [39,40]. These findings may imply that maybe a more sensitive inhibitory control task is required to detect inhibitory control differences between PTSD patients and controls, in general, and specifically in response to neutral stimuli.

The original anti-saccade task (AS; [41]) is designed to measure voluntary control of eye movements and involves inhibitory control mechanisms aimed at suppressing pre-potent response (i.e., pro-saccade, PS). Another version of the task exists that addresses both inhibitory control and cognitive flexibility; executive processes that are interrelated in attention control mechanisms [42]. In this task version, a mixed condition is present, requiring participants to switch between AS and PS responses [43]. In healthy subjects, shorter latencies of AS response were observed in switching trials as compared with the repeat trials, most likely due to high attentional resources allocation in the mixed trials [44,45]. In anxious individuals, this effect was not reported probably due to a (too) high cognitive load and impaired top-down attentional control [46]. Notwithstanding, only a few AS studies were conducted in PTSD. In a study that employed two forms of the AS task in PTSD; one with neutral standard shape stimuli and one with face stimuli, a significant group difference was evident with PTSD veterans demonstrating slowed AS latency when compared to controls in the standard AS form. In the second task which included face stimuli, PTSD veterans showed increased latency in the AS condition versus the PS condition [47]. Another study employed the AS task using eye-tracking technology. The results showed enhanced overt attentional allocation toward emotional content in PTSD, as expressed in the latencies of the first saccade in PS trials, impaired disengagement of attention from emotional content and increased RT to target identification [48].

Inhibitory control and fear inhibition (also seen in PTSD) are considered to share similar brain circuitry [49], meaning that inhibitory circuits regulate both emotional and non-emotional brain regions. Neuro-imaging studies have reported impaired inhibitory circuitry in PTSD, originating from frontal brain regions [50]. Altered anatomical and/or functional connectivity involving the frontal lobes as in PTSD ([51–60] see also reviews [30,61–63] can result in incorrect encoding of information or in the absence of encoding processes [64] and have been linked to associative memory deficits [20,21]). Associative impairments were shown to originate from an increase in FA rates (i.e., responding “yes” to a distracter in a memory recognition task; [24,65]). This can be an expression of the high cognitive load and demand of the associative test on patients’ resources (compared to the single-item recognition condition); the greater the cognitive load, the harder it becomes for PTSD patients to facilitate control over the recruitment of frontal regions, resulting in dominant automatic familiarity (rather than recollection) processes leading to increased FA rates and thus lower memory performance. Thus the current study aimed to examine to what extent inhibitory control and associative memory deficits are related in PTSD. Twenty PTSD patients and twenty-two controls were recruited to this study; all underwent a neutral non-trauma/emotional-related item-association memory task (using verbal and pictorial stimuli) and a standard AS task. Consequently, in the current study, we hypothesized that inhibitory control impairments may underlie associative memory deficits in PTSD and we aimed at testing the correlation in performance between the two tasks.

## 2. Materials and methods

### 2.1. Participants

Data collection started on 01/10/2013 and ended on 01/12/2016. Forty-two adults participated in the current study: *20* chronic-PTSD outpatients (*6* females) and *22* healthy age matched-controls (*9* females). No significant differences were found in gender distribution [*χ*^*2*^(1)=2.577, *p* = .108] or age [*t*(40)=.449, *p* = .656] between groups. Inclusion criteria (for all participants) included age > 18 years, living in Israel, and adequate proficiency in Hebrew. Exclusion criteria (for all participants) included past psychiatric (for patients, rather than PTSD) or neurological disorders and/or alcohol abuse. PTSD was diagnosed during a structured psychiatric interview using DSM-IV criteria performed by a board-qualified psychiatrist. All *20* outpatients met DSM-IV diagnostic criteria for chronic PTSD; all experienced, prior to participation in the study, a life-threatening traumatic event (e.g., car accident, work accident, terrorist attack, etc), co-morbidities were ruled out. Table 1 presents PTSD patients’ medical and trauma information. Control subjects were recruited from the community. All were Hebrew-speaking, employed and reported intact everyday functioning, In addition, all reported been in good health with no disorders and/or disabilities. The study was approved by the institutional review board of Soroka University Medical Center. All participants were Hebrew speaking and gave their written informed consent to participate in the study (in Hebrew) and were tested individually at Soroka University Medical Center.

**Table 1:** Demographics and characteristics of PTSD patients.

Subject number	Gender	Age	Months passed since the trauma	Trauma type	Medications	Body physical injury	Head physical injury
01	Male	56	60	Terror attack	Sertraline 150 mg/d, Bupropion 150 mg/d	Yes	No
02	Male	45	84	Assault	Paroxetine 30 mg/d	No	No
03	Female	54	47	Combat trauma	Citalopram 20 mg/d	No	No
04	Male	44	43	Assault	Sertraline 200 mg/d, Zolpidem 10 mg/d	No	No
05	Male	52	125	Terror attack	Escitalopram 10 mg/d	Yes	No
06	Female	44	26	Burn	Sertraline 150 mg/d,	Yes	No
07	Male	48	7	MVA	Venlafaxine 150 mg/d	No	Yes
08	Male	27	21	Work-accident	N/A	No	No
09	Male	25	20	MVA	Citalopram 40 mg/d	Yes	No
10	Female	37	240	Repeated sexual assault	Escitalopram 15 mg/d	No	No
11	Female	30	29	Combat trauma	No	No	No
12	Male	30	144	Terror attack	Mirtazapine 30 mg/d	Yes	No
13	Male	50	12	MVA	Escitalopram 10 mg/d	Yes	No
14	Male	24	7	MVA	Escitalopram 10 mg/d	Yes	No
15	Female	31	19	MVA	Duloxetine 60 mg/d	No	No
16	Male	60	492	Combat trauma	Sertraline 200 mg/d,	Yes	No
17	Male	57	12	MVA	Sertraline 150 mg/d,	No	No
18	Male	49	156	MVA	Escitalopram 10 mg/d	Yes	No
19	Female	47	36	MVA	Mirtazapine 30 mg/d	Yes	No
20	Male	38	45	Work-accident	Escitalopram 10 mg/d	Yes	No

*Note.* Data is reported for the PTSD patients group. For subject 07, an organic brain dysfunction was ruled out. MVA = Motor Vehicle Accident.

### 2.2. Measures

#### 2.2.1. Assessing PTSD symptoms.

Posttraumatic symptoms, alongside the diagnosis given by a board-qualified psychiatrist, were assessed using the Post-Traumatic Diagnostic Scale for DSM IV (PDS-4; [66,67]). This scale assesses all 17 DSM- IV PTSD symptoms, experienced in the past month. Symptoms are rated on a 4-point scale, ranging from 0 (not at all) to 3 (almost always), and the total scores ranging from 0–51. The cut-offs for symptom severity are 0 (no rating), 1–10 (mild), 11–20 (moderate), 21–35 (moderate to severe) and >36 (severe).

#### 2.2.2. Assessing memory abilities.

Memory recognition was probed using a previously published item-association memory paradigm [25,26,28,68–73]. The stimuli used in this paradigm were compiled from common Hebrew nouns of unrelated objects [74]. First, a set of 280 words was chosen, from which 120 pairs of semantically and phonologically unrelated words were created. An additional 40 words were kept to serve as distracters in the item recognition test lists (see below). Finally, for each word a black-and-white line-drawing picture was matched. In order to control stimuli type (words/pictures) 2 versions for each stimuli type were created, i.e., each stimulus was presented as a picture to half of the participants and as a word to the other half [75]. For example, half of the participants received a stimulus consisting of a picture of a house, and the other half was shown a screen on which the word ‘HOUSE’ was written. Importantly, no significant difference in the number of black pixels between each word stimulus and its’ matching line-drawing picture was found [M = 2485 ± 0.789 SD pixels and M = 2371 ± 0.1323 pixels for words and pictures, respectively; *t*(279) = 1.20; NS].

***Learning phase:*** The learning phase consisted of stimuli pairs (words/pictures), followed by item and associative memory recognition tests. Three mutually exclusive lists of 30 pairs were created. Each list contained 15 word pairs and 15 picture pairs. For balance purposes, a complementary set of 3 learning lists was created by substituting each word pair with its analogue picture pair, and vice versa (each block consisted of a learning phase, item test and an associative test). The block presentation order was randomized for each participant. During learning, participants viewed the item pairs on a 15” computer monitor, taken in random order from the study list. Stimuli were displayed, one at a time, at a rate of 4 seconds per pair (4 seconds of presentation and 1 second blank screen filler). Participants were instructed to learn both the individual items and their pairs for the upcoming item and associative recognition tests. The learning phase was followed by 30 seconds of interpolated activity (counting backwards in sevens from a randomly selected number) to eliminate the recency effect.

***Item recognition test:*** 20 stimuli were randomly presented: 10 words and 10 pictures, five of each stimulus type were targets (i.e., stimuli that had appeared in the learning list) and five were distracters (i.e., newly introduced stimuli that had not appeared in the learning phase). Participants were informed that the list included targets and distracters, and were asked to respond as quickly and as accurately as possible to each stimulus, with a designated “yes” key for targets and a “no” response key for distracters.

***Associative recognition test:*** 20 stimuli pairs were randomly presented; all had appeared during learning. The list included 10 targets (i.e., intact pairs that had appeared in the learning list) and 10 distracters (i.e., single stimuli that had appeared in the learning list but were now rearranged, thus creating new pairs). Participants were again informed that the list included targets and new-rearranged pairs, and were asked to respond as quickly and as accurately as possible to each pair, using the same keys as in the item test.

***Performance evaluation:*** Performance in both tests (item and associative) was assessed as follows: the proportion of hits (responding yes to a target) minus the proportion of FAs (responding yes to a distracter). This computation resulted in accuracy scores for each subject in each task type (item/association) and form (words/pictures). We also assessed an associative memory deficit and computed the associative deficit index (ADI). The ADI is computed from the quantitative difference between the item accuracy score and the associative accuracy score (separately for words and pictures) and it’s scores range between (−1) to (+1). Higher ADI scores represent greater associative deficit.

#### 2.2.3. Assessing inhibitory abilities.

To measure inhibitory processes we employed the anti-saccade task [41]. The AS task is a widely accepted measure of inhibitory ability [76]. Participants are asked to look toward a fixation point centered at the middle of the computer monitor. Then a visual distracter is presented on one side of the computer monitor (left/right) and is followed by a target stimulus which is quickly masked, appearing on the opposite side to the distracter. Participants are asked to respond to the target using a keyboard; for example, if the distracter appeared on the right side of the computer monitor, the target (e.g., a down arrow) appeared on its’ left side and participants are required to press the correct (down) arrow on the keyboard. The interval between the distracter and the target is called stimulus-onset-asynchrony (SOA) which varies randomly. Short SOAs are the most demanding since the pre-potent response is to look directly toward the flashing distracter, and this tendency must be quickly overcome to be able to gaze in the opposite direction and to detect the target stimulus before it disappears.

**Fig 1:**
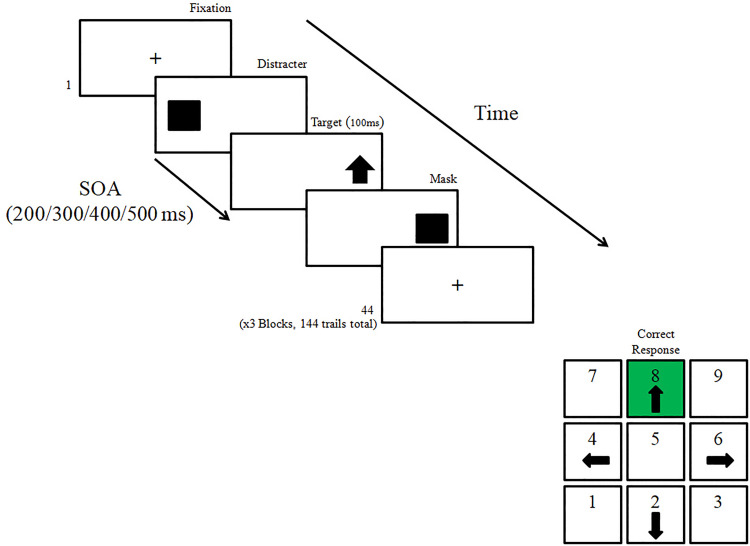
Anti-saccade paradigm. Participants are asked to look toward a fixation point centered at the middle of the computer monitor. A visual distracter was presented on one side of the computer monitor (left/right) and was followed by a target stimulus which was quickly masked in the opposite side to the distracter. Participants were asked to respond to the target using a keyboard; for example, if the distracter appeared on the right side of the computer monitor, the target (e.g., a down arrow) appeared on its’ left side and participants are required to press the correct (down) arrow on the keyboard. Stimulus-onset-asynchrony (SOA) varied and ranged between 200-500 milliseconds.

In the current study we used a modified task version [77]. Participants were instructed to fixate on a “+” which was flashed to the center of the screen. A flashing black square (distracter) was then flashed either to the left or right side of the fixation sign (visual angle corresponds to 21.4°). The SOA between the distracter (black square) and the target (an arrow pointing up, down, left or right) varied between 200–500 ms (in leaps of 100ms). The target stimulus (an arrow sign) appeared for 100 ms and was then masked by a black square until a response was recorded. All targets appeared in the opposite location of the flashing cue. Participants had to indicate the direction of the arrow (up, down, left or right) by pressing the corresponding arrow key on the keyboard. Correct answers were scored with points in order to calculate the accuracy rate. Participants in the current study performed a practice block (8 trials that were not included in the final statistical analysis) that was followed by three experimental similar blocks (44 trials each, 132 trials overall, Fig 1 presents an illustration of the task).

The total time for the procedure – introduction to the experiment, signing inform-consent, filling questionnaires, memory and the AS tasks – was approximately 50 minutes (a flowchart of the study procedure is available as supporting information, see S1 Flowchart).

#### 2.2.4. Memory performance.

To assess differences in memory performances between groups and task type, we computed a 2X2X2 three-way mixed ANOVA for accuracy rates (dependent variable) in the memory task, with *group* (PTSD/Control) as a between-subject factor and *test* (Item/Association) and *stimuli type* (Word/Picture) as within-subject factors.

### 2.3. Data transparency and openness

A power analysis using G*Power 3.1 for detecting an estimated effect size of ~0.2, an alpha level of.05 and a desired power of.9 resulted in a total sample size of 24 participants in order to detect the expected interaction effect with adequate statistical power. It should be noted that two participants from the control group did not complete the AS task, thus results for this task and relevant correlation analysis are reported for n = 20 participants in this group. Analysis of variance was conducted using STATISTICA software (version 12), an advanced analytics software package originally developed by StatSoft and currently maintained by TIBCO Software Inc. Correlations and regression analysis were conducted using IBM Statistical Package for Social Sciences (SPSS, version 25). The data that support the findings of this study are available in: https://osf.io/wg5yp/. This study was not preregistered.

## 3. Results

### 3.1. PTSD symptomatology

A significant difference in the total PDS score was found between PTSD patients and controls (M_PTSD_ = 34.9 ± 12.876, M_controls_ = 21.5 ± 4.848; [*t* (40) = 4.381, *p* = .000]. Significant differences in the PDS-sub scales were also found; these include the re-experiencing sub-scale (M_PTSD_ = 7.2 ± 5.736, M_controls_ = 1.727 ± 2.353; [*t* (40) = 4.113, *p* = .000]), the avoidance sub-scale (M_PTSD_ = 9.55 ± 7.632, M_controls_ = 1.454 ± 2.303; [*t* (40) = 4.747, *p* = .000] and the arousal sub-scale (M_PTSD_ = 7.2 ± 5.463, M_controls_ = 2.045 ± 2.802; [*t* (40) = 3.99, *p* = .000].

**Fig 2:**
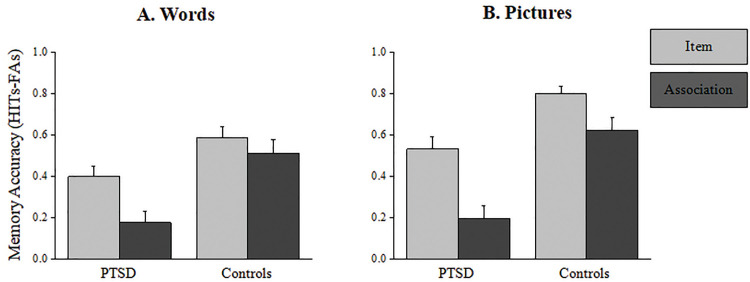
Memory accuracy. Item and associative memory accuracy for words (A) and pictures **(B)**. Error bars represent the standard error of the mean.

The statistical analysis revealed a significant main effect for *group*, PTSD patients showed lower memory accuracy compared to control subjects (M_PTSD_ = .328 ± .051, M_controls_ = .632 ± .048; [*F* (1, 40) = 18.853, *p* = .000, *η*_*p*_^*2*^ = .32]. A second main effect was found for *test*, item recognition was significantly higher than associative recognition (M_item_ = .581 ± .033, M_association_ = .378 ± .042; [*F* (1, 40) = 55.161, *p* = .000, *η*_*p*_^*2*^ = .58]. A third main effect was found for *stimuli type*, recognition of pictures was significantly higher than recognition of words (M_pictures_ = .539 ± .036, M_words_ = .42 ± .038; [*F* (1, 40) = 23.279, *p* = .000, *η*_*p*_^*2*^ = .368]. A two-way interaction was found between *stimuli type* and *test* [*F* (1, 40)=8.803, *p* = .005, *η*_*p*_^*2*^ = .18], with pictorial stimuli significantly enhancing item memory [*F* (1, 40)= 33.038, *p* = .000, *η*_*p*_^*2*^ = .45] but not associative memory [*F* (1, 40)= 4.209, *p* = .046, *η*_*p*_^*2*^ = .095]. A second two-way interaction was found between *group* and *test* [*F* (1, 40)= 7.651, *p* = .009, *η*_*p*_^*2*^ = .161]. This interaction showed lower associative memory performance in the PTSD group compared with item memory [*F* (1, 40)= 49.588, *p* = .000, *η*_*p*_^*2*^ = .553]; the same pattern was observed in the control group and was significant but smaller [*F* (1, 40)= 11.405, *p* = .002, *η*_*p*_^*2*^ = .222]. The two-way interaction between *group* and *stimuli type* was close-to-significance [*F* (1, 49)= 2.981, *p* = .092, *η*_*p*_^*2*^ = .069]. The three-way interaction was not significant [*F* (1, 40)=.01, *p* = .922, *η*_*p*_^*2*^ = .00] (see Table 2 and Fig 2).

**Table 2:** Means and Standard Errors for Hits and False Alarms (FA) for item and associative recognition of Words and Pictures.

		PTSD		Controls	
		Item	Association	Item	Association
	%Hit	0.595 (0.033)	0.561 (0.031)	0.754 (0.040)	0.765 (0.038)
Words	%FA	0.195 (0.038)	0.383 (0.041)	0.166 (0.035)	0.251 (0.046)
	%Hit - %FA	0.400 (0.051)	0.1783 (0.056)	0.587 (0.055)	0.513 (0.066)
	%Hit	0.716 (0.041)	0.675 (0.044)	0.866 (0.024)	0.877 (0.025)
Pictures	%FA	0.183 (0.033)	0.476 (0.042)	0.063 (0.026)	0.254 (0.052)
	%Hit - %FA	0.533 (0.060)	0.198 (0.063)	0.803 (0.036)	0.622 (0.063)

*Note.* FA = False Alarm. Hits and FAs are shown in absolute values while the sensitivity measure (%Hits-%FAs) is standardized between (−1.00) to (+1.00).

To specifically address an associative memory deficit (defined as the difference between item recognition and associative recognition performance), we compared the ADI results of the PTSD patients and the control participants. This analysis revealed a significant difference between the groups, PTSD patients showed higher ADI scores compared to controls [M_PTSD_ = .222 ± .238, M_controls_ = .074 ± .175; [*t* (40) = 2.299, *p* = .027; M_PTSD_ = .34 ± .204, M_controls_ = .18 ± .232; [*t* (40) = 2.284, *p* = .028; for words and pictures respectively]. Of note, higher ADI scores can result from a decrease in hits rates, or an increase in FA responses, or both. To assess the locus of the associative deficit we computed a four-way 2x2x2x2 mixed ANOVA with *group* (PTSD/Control) as a between-subject factor and *test* (Item/Association), *stimuli type* (Word/Picture) and *response* (Hit/FA) as within-subject factors. This analysis revealed a significant two-way interaction between *test* and *response* [*F* (1, 40) = 55.161, *p* = .000, *η*_*p*_^*2*^ = .58]. Planned comparison analysis showed no significant difference between tests on hits responses [*F* (1, 40) =.667, *p* = .419, *η*_*p*_^*2*^ = .01] but a significant difference in FA responses [*F* (1, 40) = 53.326, *p* = .000, *η*_*p*_^*2*^ = .57]. Furthermore, the interaction between *test* X *response* X *group* was significant [*F* (1, 40) = 7.651, *p* = .008, *η*_*p*_^*2*^ = .16]. Planned comparison analysis showed that the simple interaction between *test* and *response* was significantly stronger in the PTSD group as compared to the control group [[*F* (1, 40) = 49.588, *p* = .000, *η*_*p*_^*2*^ = .55] and [*F* (1, 40) = 11.405, *p* = .002, *η*_*p*_^*2*^ = .22]]. Further analysis showed that in both groups there was no significant difference between hIT responses [[*F* (1, 40) = 2.477, *p* = .123, *η*_*p*_^*2*^ = .06] and [*F* (1, 40) =.217, *p* = .643, *η*_*p*_^*2*^ = .00]; for PTSD and controls, respectively], while a sharp difference in FA responses was found in the PTSD group [*F* (1, 40) = 41.170, *p* = .000, *η*_*p*_^*2*^ = .51] compared to the control group [*F* (1, 40) = 14.843, *p* = .000, *η*_*p*_^*2*^ = .27]. This pattern confirms that the associative deficit in the PTSD group is due to an increase in FA responses.

To assess differences in RT, we computed a three-way 2x2x2 mixed ANOVA, with *group* (PTSD/Control) as a between-subject factor and *test* (Item/Association) and *stimuli type* (Word/Picture) as within-subject factors, and the dependent variable as response latency. The statistical analysis revealed three significant main effects: for *group*, *test* and *stimuli type*. Overall, the PTSD patients presented longer latencies compared to control subjects (M_PTSD_ = 3225.274 ± 239.547, M_controls_ = 2127.980 ± 228.399; [*F* (1, 40) = 10.991, *p* = .002, *η*_*p*_^*2*^ = .216]. Response latency in the items condition was shorter than for the associative condition (M_item_ = 2212.977 ± 160.713, M_association_ = 3140 ± 182.584; [*F* (1, 40) = 97.912, *p* = .000, *η*_*p*_^*2*^ = .71]. Lastly, response latency for pictures was shorter than for words (M_pictures_ = 2566.943 ± 179.129, M_words_ = 2786.311 ± 163.028; [*F* (1, 40) = 6.184, *p* = .017, *η*_*p*_^*2*^ = .134]. No interaction effects were found.

### 3.2. AS performance as a measure of inhibition

To assess the difference in inhibitory abilities between groups we computed a two-way mixed ANOVA to accuracy levels with *group* (PTSD/Control) as a between subject factor and *SOA* (200, 300, 400, 500 ms) as a within subject factor.

**Fig 3:**
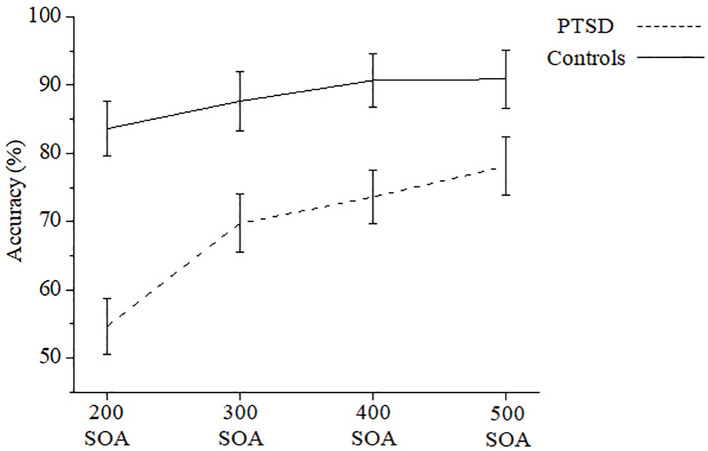
Inhibitory performance. Accuracy is presented for 200-500 stimulus-onset-asynchrony (SOA), for PTSD patients and controls. Error bars represent the standard error of the mean.

The statistical analysis yielded a significant main effect for *group*, with PTSD patients showing lower accuracy as compared to controls (M_PTSD_ = 70.177 ± 22.869SD, M_controls_ = 88.209 ± 7.991SD; [*F* (1, 38) = 12.680, *p* = .001, *η*_*p*_^*2*^ = .250]). A second main effect was found for *SOA*, with longer SOAs showing higher accuracy rates (M_200_ = 69.146 ± 23.187SD, M_300_ = 78.701 ± 20.986SD, M_400_ = 82.154 ± 19.467SD, M_500_ = 84.51 ± 19.789SD; [*F* (3, 114) = 26.070, *p* = .000, *η*_*p*_^*2*^ = .407]). Planned-comparisons analysis showed a significant difference in accuracy between the 200ms SOA and 300ms SOA, [*F* (1, 38) = 24.743, *p* = .000, *η*_*p*_^*2*^ = .394]; between the 300ms and 400ms SOA [*F* (1, 38) = 6.057, *p* = .018, *η*_*p*_^*2*^ = .137], and 500ms SOA and all the other SOAs conditions, [*F* (1, 38) = 36.661, *p* = .000, *η*_*p*_^*2*^ = .491]. Lastly, the interaction between *group* and *SOA* was significant (*F* (3, 114) = 6.810, *p* = .000, *η*_*p*_^*2*^ = .152). Planned-comparisons analysis showed a reduced group effect as the SOA increases [*F* (1, 38) = 25.356, *p* = .000, *η*_*p*_^*2*^ = .400; *F* (1, 38) = 8.717, *p* = .005, *η*_*p*_^*2*^ = .186; *F* (1, 38) = 9.323, *p* = .004, *η*_*p*_^*2*^ = .196; *F* (1, 38) = 4.540, *p* = .039, *η*_*p*_^*2*^ = .107; for 200, 300, 400, 500 SOA, respectively], see Table 3 and Fig 3.

**Table 3:** Means and standard deviations for PTSD and controls in the anti-saccade task.

		PTSD	Controls
Accuracy (%)	200 SOA	54.66 (21.99)	83.63 (13.34)
	300 SOA	69.75 (25.50)	87.65 (9.20)
	400 SOA	73.62 (23.52)	90.68 (8.43)
	500 SOA	78.12 (25.83)	90.89 (7.09)
	total	70.17 (22.86)	88.20 (7.99)
Reaction Time (RT, ms)	200 SOA	1232.06 (767.37)	937.71 (775.73)
	300 SOA	1112.84 (643.08)	940.79 (935.91)
	400 SOA	1116.18 (665.68)	748.85 (232.96)
	500 SOA	1039.37 (645.40)	738.29 (224.91)
	total	1113.20 (669.07)	841.24 (504.79)

*Note.* SOA = stimulus-onset-asynchrony.

To assess differences in RT between groups and SOA, we computed a two-way ANOVA, with Group (PTSD/Control) X SOA (200, 300, 400, 500 ms) as independent factors, and the dependent variable as response latency. This statistical analysis yielded a non-significant main effect for *group*, (M_PTSD_ = 1113.204 ± 669.077 SD, M_controls_ = 841.245 ± 504.790 SD; [*F* (1, 38) = 2.334, *p* = .135, *η*_*p*_^*2*^ = .058]. A significant main effect was found for *SOA*, (M_200_ = 1084.889 ± 776.058 SD, M_300_ = 1026.820 ± 797.372 SD, M_400_ = 932.519 ± 526.233 SD, M_500_ = 888.830 ± 500.823 SD; [*F* (3, 114) = 2.768, *p* = .045, *η*_*p*_^*2*^ = .068]). Planned comparison analysis showed significantly faster response latencies from 200ms to 500ms [*F* (1, 38) = 5.414, *p* = .025, *η*_*p*_^*2*^ = .125], all other comparisons did not reach significance [*p* > .05]. The two-way interaction did not reach significance (*F* (3, 114) = 0.58, *p* = .629, *η*_*p*_^*2*^ = .015).

### 3.3. Relationships between PTSD severity, memory and inhibition

To assess the relationship between PTSD severity and each of the cognitive measures evaluated in the current study (memory, inhibition), Pearson correlation coefficients were computed. PTSD severity (as assessed by the total PDS score and its sub-scales; re-experiencing, avoidance and hyper-arousal) in the patients group was negatively correlated to inhibitory performance as assessed by the total accuracy score in the AS task (*r* = −.5771, *p* = .008; *r* = −.6117, *p* = .004; *r* = −.5563, *p* = .011; *r* = −.5994, *p* = .005, respectively). Trends were found between PTSD severity and RT in the AS task (*r* = .405, *p* = .076 for total PDS score; *r* = .424, *p* = .062 for hyper-arousal). No significant correlations were found between the total PDS score (and/or its sub-scales) and inhibitory performance in the control group.

Regarding memory, no significant correlations were found between the total PDS score (or its sub-scales) and memory accuracy in any of the groups, however, a significant positive correlation was found between the PDS hyper-arousal severity score and FA rates in the item-words recognition measure in the PTSD group (*r* = .457, *p* = .043). Trends in the same direction were found for the re-experiencing sub-scale (*r* = .403, *p* = .078), the avoidance sub-scale (*r* = .394, *p* = .085) and total PDS score (*r* = .424, *p* = .062).

**Fig 4:**
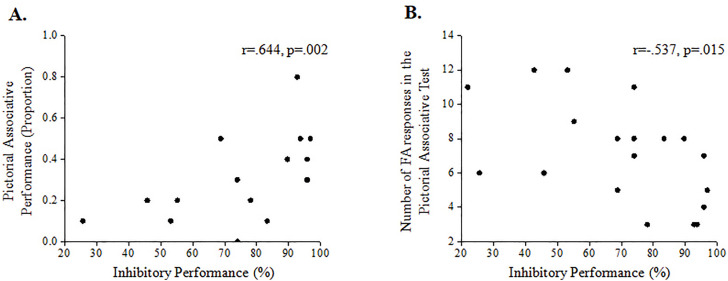
Correlation analysis results for memory and inhibition measures. Inhibitory performance was positively correlated to pictorial associative recognition (A) and negatively correlated to the number of false-alarm (FA) responses (B) in PTSD patients.

In order to test the hypothesis regarding the role of inhibitory abilities in memory performance, two correlation matrixes were computed, for words and pictures separately. Since no significant correlations were found in the control group, results are reported for the patients group only. No significant correlations were found between inhibitory performance and verbal/words memory performance in PTSD patients; a trend was found between inhibitory performance and verbal memory recognition of items (*r* = .423, *p* = .063). Pictorial memory performance correlated to inhibitory performance (see Fig 4); a significant positive correlation between associative pictorial recognition and inhibitory performance was found (*r* = .644, *p* = .002) and accordingly, a significant negative correlation between the number of FA responses in the associative pictorial test and inhibitory performance was also found (*r* = −.537, *p* = .015). Similar to the findings in words, a trend was found between inhibitory performance and pictorial memory recognition of items (*r* = .434, *p* = .055).

## 4. Discussion

The aim of the current study was to examine the relationship between inhibitory control and associative memory deficits in PTSD. Specifically, we hypothesized that inhibitory control impairments may underlay associative memory deficits in PTSD. Using a neutral, non-trauma/emotional-related item-association memory task and the AS task we showed that generally, the performance of PTSD patients’ was lower than the controls’ performance in both tasks. Regarding memory, we used a pixel-balanced item-association memory task and replicated previous findings by showing lower associative (as compared to item) memory performance in the PTSD group (as compared to controls); this pattern was attributed to increased FA responses in this group. Most importantly, we confirmed the relationship between inhibitory control impairments and (pictorial) associative memory deficits in PTSD by showing a strong significant positive correlation between associative pictorial memory performance and inhibitory performance, and accordingly, a significant negative correlation between the number of FA responses in the associative pictorial test and inhibitory performance.

The findings of the current study correlate with previous work showing associative memory deficits in PTSD patients [22,24–26], healthy controls under stress [68] and negative emotional arousal manipulations [69]. The current study findings also replicate previous results confirming that the locus of the associative deficit in PTSD lies in increased FA rate in this group [24]. This increase can result from altered frontal anatomical and/or functional connectivity as previously reported in PTSD [51–63]. Altered frontal connectivity during memory tasks may lead to the wrong encoding of information or to the absence of encoding processes [64] and thus in turn, to low memory accuracy. The results of the current study support this view by showing that encoding in PTSD is inefficient, as a high FA rate means that patients rely on familiarity (rather on recollection) processes during episodic memory tasks. This is especially dominant in the associative condition which is cognitively demanding (as compared to the single-item recognition condition) and involves high cognitive load and executive control, both of which have been reported to alter in PTSD [30].

In the current study, associative memory deficits in PTSD, resulting from increased FA responses, were thought to involve impaired inhibitory control. Whereas differences in inhibitory control between PTSD patients and healthy participants exist, findings are mixed and heterogeneous [34–40,47,48,78]. This heterogeneity led us to probe inhibitory control using the AS task, which is designed to measure inhibitory control resources aimed at suppressing a pre-potent response. The motivation to use this task arose from observations that showed that PTSD patients show decreased inhibition to fear responses even in a safe environment [79–81]; the over-generalization tendency of posttraumatic patients to respond to neutral cues, as if they were traumatic cues, leads to elevated FA rates responses that initiate an escalation of traumatic symptoms. Since inhibitory control and fear inhibition are considered to share similar brain circuitry [49,50], in the current study we hypothesized that the failure of inhibitory control mechanisms underlies both clinical symptoms and memory impairments in PTSD.

In the current study, inhibitory performance and PTSD clinical severity were related; a significant negative correlation was observed between inhibitory performance and symptom severity in PTSD, i.e., high symptomatic patients presented lower inhibitory performance. This finding corresponds with studies pointing toward inhibitory control impairments that may predate the traumatic exposure, serve as potential risk factors for PTSD development and predict symptoms severity [30,34,82–84]. Recently, response inhibition training was shown to correlate with an improvement in PTSD symptoms from pre-to post-training, compared to the control condition [85]. In addition, inhibitory performance significantly correlated to both pictorial associative performance and the number of FA responses under the pictorial associative condition (i.e., the locus of the associative deficit) in PTSD patients. This finding may imply that, normally, inhibitory resources are required for correct associative recognition; these resources facilitate stronger dominant recollection processes that enable individuals to judge whether the presented items were presented together (or not) during the learning phase based on specific details. Notwithstanding, these recollection processes require executive functioning, which is known to be impaired in PTSD. In this manner, the significant correlation between inhibitory performance and associative memory performance in the PTSD group can be attributed to low inhibitory resources in this group, high FA rates leading to lower associative memory accuracy.

Several limitations of the current study must be acknowledged. Firstly, due to its cross-sectional nature, we cannot determine whether inhibitory control deficits are a pre-existing predisposition or an outcome of traumatic exposure and/or PTSD. Although several studies support the former [34,82–84], future prospective-longitudinal studies are needed to answer this question. These studies can benefit from use mediation analysis to strengthen the claim that inhibitory failures underlie the associative memory deficits exhibited by PTSD patients. Secondly, although there is a consensus that PTSD patients show impairments when they are asked to withhold responses [86], most studies have used inhibitory control tasks such as the Go/No-Go or Stop-Signal and only a few of them probed inhibition using the AS task; these works to the best of our knowledge did not link inhibitory impairments to other cognitive domains (e.g., memory). Thus, in order to generalize the results of the current study, replication using similar/different inhibitory measures and reference to other cognitive functions, is required.

Understanding the mechanisms through which inhibitory failures and associative deficits occur in PTSD is carries highly important clinical implications as these influence daily functioning and treatment. Memory deficits reduce available coping resources and can impact patients’ ability to engage in and respond to psychological treatment. This view is supported by empirical studies showing that verbal memory impairments predicted poorer treatment outcomes in PTSD patients receiving cognitive behavioral therapy [87] and psychotherapy [88]. With appropriate caution, our findings suggest that PTSD patients may benefit from interventions focusing on improving inhibitory abilities. This improvement, if present, is expected to lead to better associative memory abilities in PTSD patients which will hopefully increase their daily functioning and improve their engagement to treatment. Clinically, an improvement in inhibitory abilities is expected to reduce intrusive symptoms and thus improve patients’ condition. Due to the growing recognition of the importance to evaluate both individual and group-level treatment responses in PTSD [89,90], we suggest that future studies should examine the possible predictive value of different cognitive neuropsychological profiles, with an emphasis on memory and inhibitory abilities, in rehabilitation results for PTSD.

## Supporting information

S1 Flowchart A flowchart of the study procedure. (DOCX)
