# Supplementary material for: Inhibitory control impairments underlie associative memory deficits in posttraumatic stress disorder
Source: PLoS One. 2025 Aug 14;20(8):e0329810. doi: 10.1371/journal.pone.0329810 (PMC12352785; doi:10.1371/journal.pone.0329810)
Supplement: Supplementary file 1 [file pone.0329810.s001.docx]

PTSD Symptom Questionnaire (PDS-4)

Item-Association Memory Task

For Pictures and Words (Mixed Block)

Learning Phase

Item

Memory 

Test

Associative Memory 

Test

Anti Saccade Task

X3

Blocks

X3

Blocks
